# Features of urban green spaces associated with positive emotions, mindfulness and relaxation

**DOI:** 10.1038/s41598-022-24637-0

**Published:** 2022-11-30

**Authors:** Agnieszka Olszewska-Guizzo, Angelia Sia, Anna Fogel, Roger Ho

**Affiliations:** 1 Institute for Health Innovation & Technology (iHealthtech), National University of Singapore, Singapore, Singapore; 2 NeuroLandscape Foundation, Warsaw, Poland; 3 National Parks Board, Centre for Urban Greenery and Ecology, Singapore, Singapore; 4 Department of Psychological Medicine, Yong Loo Lin School of Medicine, National University of Singapore, Singapore, Singapore; 5 Singapore Institute for Clinical Sciences, Agency for Science, Technology and Research, Singapore, Singapore

**Keywords:** Urban ecology, Psychology, Quality of life, Sensory processing, Stress and resilience, Environmental impact, Environmental social sciences

## Abstract

There is an established consensus among researchers that contact with nature improves mental health, wellbeing, and quality of life in urbanised environments. Studies tend to examine the health impacts of nature without identifying specific physical and spatial landscape features that could guide health-promoting design of urban green spaces. A growing body of evidence suggests that landscape features described in the Contemplative Landscape Model (CLM) can be used to measure therapeutic value of urban landscapes. CLM assesses urban landscapes across seven sub-scales: *Layers of the Landscape, Landform, Vegetation, Color and Light, Compatibility, Archetypal Elements* and *Character of Peace and Silence*. We exposed 74 healthy adults to six urban landscapes in laboratory (video representations) and naturalistic outdoor settings. We explored the associations between the visual quality of urban landscapes annotated with CLM, with self-reported positive emotions and brain activity consistent with mindfulness (Theta waves), relaxation (Alpha waves) and attention restoration (Beta waves), and differences between laboratory and naturalistic setting. CLM scores predicted self-reported Valence and Arousal, and low frequency power bands: Alpha and Theta in the naturalistic setting. Landscape features showing the strongest associations were *Character of Peace and Silence, Layers of the Landscape* and *Archetypal Elements.* Alpha, Theta brain reactivity and Arousal scores, were significantly different between laboratory and naturalistic settings (*p* < 0.05), while Valence scores between those settings were statistically identical (*p* = 0.22). Self-reported Valence and Arousal, but not brain activity, were significantly associated with the majority of landscape features in the laboratory setting. The results of the study provide guidelines on the urban landscape features most beneficial for human health, to inform urban green space design.

## Introduction

It has been well-established that the benefits of exposure to nature scenes go beyond aesthetic appreciation, extending to cognitive, affective as well as mental and physical health benefits (for detailed review see^1^). Mental health benefits of greenspaces have been supported by multiple studies synthesized in several systematic reviews (e.g.,^2–4^), but the causal pathways have not been fully established. One of the leading theories is Attention Restoration Theory, which proposes that greenspaces help replenish depleted attention capacity through experiencing natural settings^5,6^, thus promoting wellbeing. The complementary Stress Reduction theory^7^ suggests that natural environments promote recovery from stress, while the Biophilia hypothesis offers a somewhat philosophical explanation, which assumes an intrinsic affection towards unthreatening nature, shaped by the evolution^8^. With the rapid urbanization leading to overstimulating living environments, natural environments give an opportunity for attention restoration through “being away” from the city noise^9^. In the face of global decline in mental health^10^, city inhabitants are at higher risk of developing mental disorders such as depression and anxiety than their rural counterparts^11,12^. Therefore, nature in city, also known as urban green spaces (UGS), nature-based solutions, green infrastructure or simply urban parks and gardens, can be a promising medium to offset the negative mental health outcomes associated with living in high-density cities.

The challenge that landscape architects and urban planners are facing is the lack of evidence-based guidelines to include in their UGS designs and maintenance plans, to promote positive affect, mental health and well-being. Importantly, there is a clear lack of typology of UGS based on their associations with mental health promotion^13^. Previous research on UGS for health and wellbeing predominantly compared the effects of *urban* versus *nature* conditions, where “nature” had rather broad definition—a landscape containing natural elements for example trees, water, grass (e.g.^14^), or simply focused on the quantity of greenspace as measured with aerial photography techniques, (e.g.^15^). Both approaches offer little or no implications for landscape architectural design as perceived by people. Other studies used different UGS typologies as comparators with the urban space, for example forest (e.g.^16^), park (e.g.^17^), garden (e.g.^18^). However, these approaches seem to leave too much room for ambiguity as there is a myriad of landscape scenes and physical attributes within each of the UGS type, different styles of design and quality of maintenance, likely providing different levels of salutogenic potential. The inconsistencies in findings across the studies suggest that a more granular analysis of the landscape features is required to help inform health promoting urban greenspace designs. The lack of specific knowledge on the landscape quality in the area of evidence-based landscape design has been highlighted by researchers^19,20^.

Another issue lies with the methodological aspects of existing studies, more specifically their ecological validity and replicability. A vast majority of studies have been conducted with photographic representations of nature, in the laboratory environment (e.g.^21^), or only in the outdoor setting (e.g.^22^). Trials in the controlled laboratory settings have the advantage of fewer confounding variables, particularly since participant responsiveness to the real in-vivo landscape setting and the corresponding photograph of such setting are highly correlated. Nevertheless, that important sensory-cognitive factors are excluded in laboratory-based experiments thus may not have the same effect on the participants^23^. Further research is needed to compare the associations between landscape exposure and participant outcomes in both laboratory and naturalistic settings. This leads to a need for systematic methods of evaluation of the visual landscape quality and/or features, taking into account the complexity and the dynamic character of living landscapes. The existing frameworks include the Scenic Beauty Estimation method^24^ and Visual Resources Management tool^25^ developed for evaluation and management of the vast areas of natural parks, however, their applicability in dense, urban landscapes is limited. Elements of the urban visual quality assessment can be found in more modern frameworks including Urban Landscape Quality Index^26^ or RECITAL^27^, which have been developed and tested, however have not been calibrated specifically for UGS and not yet extensively validated.

**Figure 1:**
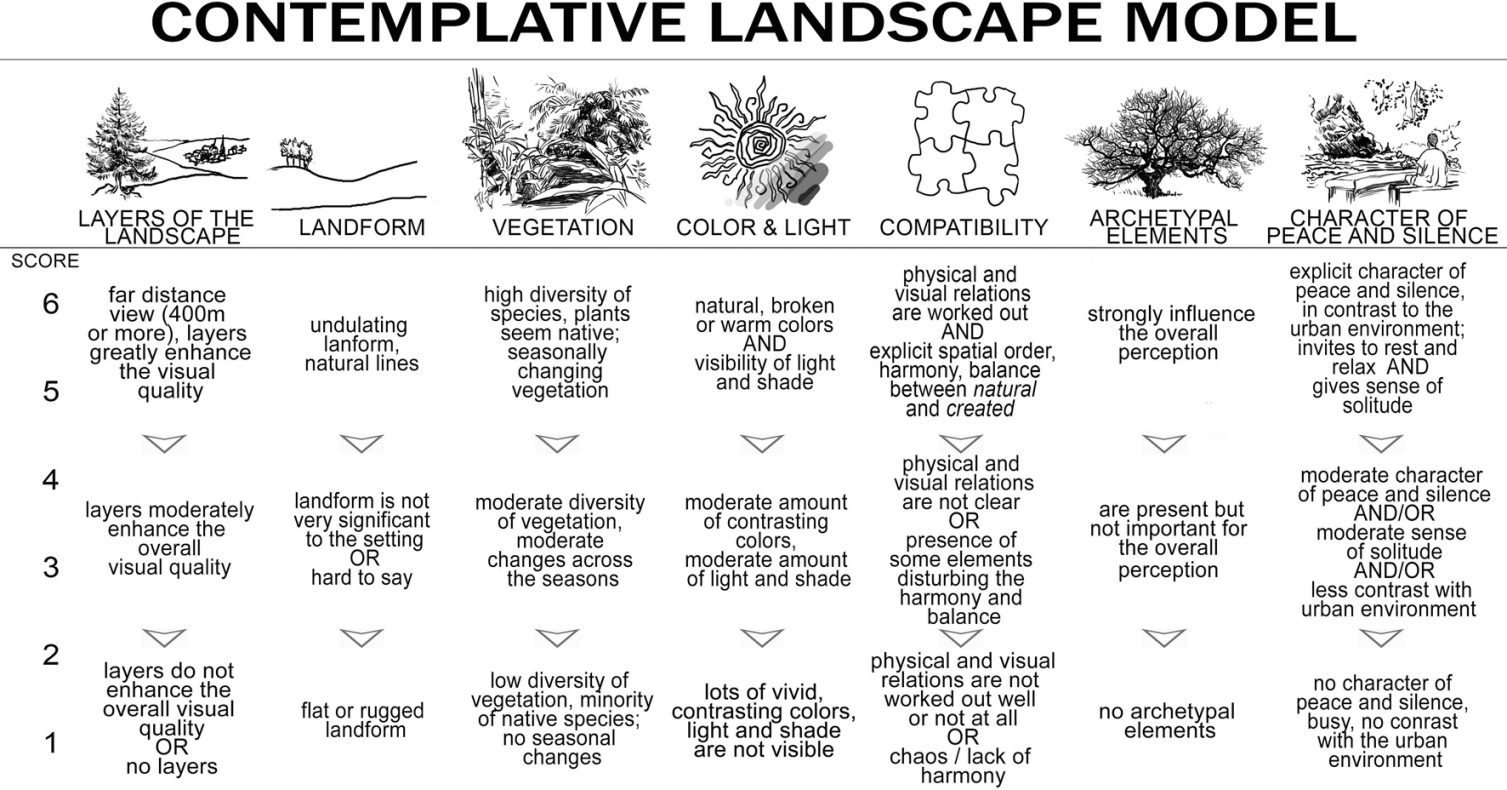
Contemplative Landscape Model with seven key-components and scoring system based on 1–6-point scale. Adapted from^32^.

Contemplative Landscape Model (CLM), is a validated, expert-based UGS assessment instrument with scope of mental-health and well-being promotion. CLM borrows from the previous visual quality assessment methods^28,29^, traditions of landscape design theory^30^ as well as insights from Jungian psychoanalysis^31^. According to the CLM, each UGS view can be scored according to seven key-categories (see Fig. 1): *Layers of the Landscape*—assessing the depth of the view and possibility of noticing fore, middle and background in the scene; *Landform*—focusing on the natural asymmetry of the topography and characteristics of the skyline—whether the landscape stimulates our eyes to look upwards; *Vegetation*—scenes rich in species with plants that seem self-sown and not overly tended and subject to changes along the daily/seasonal/life cycle; *Color & Light*—scoring the possibility of seeing light and shade movement casted on the ground, view-point away from direct sun exposure as well as presence of less saturated colors; *Compatibility—*assessment of harmony and balance of the composition of the scene, and absence of distracting or incompatible elements; *Archetypal Elements—*explicit presence of elements of the landscape loaded with symbolic and universal meaning (e.g. waterfall, single tree, stone), and *Character of Peace and Silence—*assessment of the potential for resting, comfort and a sense of solitude, offering contrast to the busy urban space. CLM allows the assessment of individual features of urban landscapes to inform design.

Further research is needed to improve our understanding on the effects of features of UGS on human health and well-being, as well as individual differences in responses to various UGS. Furthermore, it is unclear whether there are differences in participant responses to landscapes presented in the laboratory conditions vs in-situ naturalistic exposure. Finally, a deeper understanding of the cognitive processes that mediate the effects of UGS on health and well-being, using established objective measures is necessary to elucidate causal mechanisms.

The aim of this study was to investigate whether CLM predicts patterns of brain activity associated with attention restoration (beta), wakeful relaxation (alpha) and mindfulness (theta), and self-reported affect (Valence and Arousal). And, what specific CLM features are associated with these positive outcomes. Moreover, to explore the similarities and differences in participant responsiveness to landscapes in laboratory and naturalistic settings.

## Methods

### Participants

We recruited 79 healthy adults, 48 were female, with age ranging between 21 and 74 years old. The participants were recruited using snowball sampling methodology. The inclusion criteria were age between 21 and 75 years old, right handedness (due to differences in brain activity between left and right-handed people^33^) and availability to attend the required study visits. The exclusion criteria were serious visual impairment or clinically diagnosed psychiatric, neurological, or cognitive disorders. All participants were reimbursed for their time. Procedures were reviewed by the National University of Singapore Ethics Committee and obtained ethics approval, NUS-IRB_S-20-12, and experiments were performed in accordance with relevant guidelines and regulations. All participants provided informed consent for taking part in the study.

### Psychometric tools

#### Contemplative Landscape Model (CLM)

Landscape views were scored according to seven CLM categories on 1–6-point scale, and the total score for a view is calculated by averaging all categories scores by 4 independent experts in landscape architecture. CLM is an expert-based psychometric tool with good reliability (Cronbach’s alpha = 0.854) and validity measured by correlation with the validating set of data (*r* = 0.772)^32^. In one existing laboratory-based within subjects neuroscience experiment, highly contemplative landscapes (above 4.45 points) induced a statistically different pattern of brain activity as compared to less contemplative views, in the brain of healthy adults passively exposed to them^34^.

#### Self-Assessment Manikin (SAM)

This tool was used to record the self-reported affective response of participants, after exposure to each landscape scene in the laboratory as well as the naturalistic setting. SAM is a non-verbal, pictorial assessment of momentary emotions. In our study we used two SAM scales to measure Valence (i.e., pleasantness) and Arousal (i.e., intensity of emotion)^35^. The instrument consists of five pictograms with facial expressions for Valence and five for Arousal and ranged from − 2 to 2 points. A higher Valence score indicates more positive emotion towards the stimuli and a higher Arousal score indicates higher intensity of that emotion towards the stimuli.

#### Beck Depression Inventory (BDI-II)

This instrument was used at the start of the study, to assess the depression levels of participants. This 21-item multiple choice self-reported questionnaire measures the severity of depression, and is often used by psychiatrists due to its high sensitivity (81%) and specificity (91%)^36^. Questions refer to feelings experienced during the two weeks prior to and including the assessment day. Subjects were to circle one of four to seven statements under each of 21 items, which have assigned scores of 0, 1, 2 or 3 points. To compute total BDI-II score points from all items are summed up. Total score of 0–13 denotes minimal depression; 14–19, mild depression; 20–28, moderate depression and 29–63, severe depression.

### Neuroscience tools

Electroencephalography (EEG) signal was recorded using a 16-channel V-amp amplifier (Brain Products GmbH, Munich, Germany) equipped with dry active electrodes mounted on an elastic cap according to the modified 10/20 system. The fact that dry electrodes were selected was especially meaningful for the outdoor scans—using wet electrodes and applying gel outdoors under the hot and humid climate of Singapore could reduce participants’ comfort and significantly increase the time of experiment. Electrode impedance was kept below 100 kΩ throughout the experiment, which is considered an acceptable value for dry electrodes^37^. Signal was recorded at 500 Hz and stored for further processing. The following indices were utilised to evaluate the results of EEG scans:

#### Frontal alpha activity—relaxation

Alpha rhythm (8–13 Hz) in awake state is typically strongest when individuals are not actively engaged in cognitive tasks. Alpha waves are often considered to reflect cortical “idling”, with a reduction in its power when the individual attends actively to stimuli and/or undertake a cognitive task. Thus, magnitude of the band is inversely proportional to cortical activation^38^. Increased alpha power in the frontal cortex is associated with lower level of psychological and emotional arousal, akin to wakeful relaxation^39^. Previous environmental neuroscience studies found the increased frontal alpha power in participants exposed to nature scenes as compared to urban scenes^40^, and in environments with less stressful conditions^41^. This pattern of brain activity is especially important for city dwellers as it can be contributing to reduction of stress and burnout and increasing life satisfaction (e.g.^42^). In our experiment we averaged alpha power from three pairs of electrodes located on the frontal lobes (AFp1-AFp2; AFF5h-AFF6h and F7-F8) to obtain the Alpha/Wakeful Relaxation index.

#### Frontal theta activity—mindfulness

Theta rhythm (3–7 Hz) is often related to cognitive processing in prefrontal cortex^39^. At the same time, increased relative theta power was associated with physiological relaxation^43,44^ and high presence in the moment^45^. Therefore, researchers relate the increased frontal theta power with meditative state of a non-directive and non-concentrative style^46,47^. The concentrative meditation (e.g., Transcendental) aims to eliminate the thoughts through intense focus on a single stimulus (e.g., mantra), while non-directive meditation (e.g. Mindfulness) allows thoughts to flow freely, without focusing on anything that arises internally. Mindfulness, by its psychology definition, is associated with being attentive to the present moment (sometimes referred to as “hyper-presence”) and lack of interpretation or judgement of the experienced phenomena^48^ without cognitive processing of external stimuli. Inducing this state in brain is increasingly recognized as a self-care and wellness intervention to reduce stress^49^, with range of mindfulness-based therapies and interventions targeting various mental health disorders^50^. Previous research in environmental neuroscience associated frontal theta brainwave pattern with the walk in nature^51^ and the area satisfaction^52^. The mindfulness index for this study was calculated by averaging Theta power from three pairs of electrodes located on the frontal lobes (AFp1-AFp2; AFF5h-AFF6h and F7-F8).

#### Temporal beta asymmetry—attention restoration

Beta rhythms (14–30 Hz) mark attentional processing and are increased during task engagement. Temporal Beta Asymmetry is characterized by more beta power in the right temporal lobe than in the left. The temporal region of the right hemisphere are, among other functions, responsible for visual attention^53^, interpreting visual information and memory of pictures, visual scenes and familiar faces^54^. Previous studies associated this pattern of brain activity with bottom-up, stimuli driven attention directed at the salient stimuli^55^. This bottom-up type of attention is triggered by the external stimuli, and opposite to goal—oriented attention, typical to processing of the task, which, when performed for too long, leads to mental fatigue. Bottom-up attention is the central concept of the Attention Restoration Theory, according to which contact with the natural environments is considered a triggering factor leading to restoration of depleted attention capacity and recovery from mental fatigue. In previous studies attention restoration was conceptually linked to ‘fascination'—a key-component of restorative environments, according to ART^56,57^. The Attention Restoration Index in this study was computed with Beta power values from the left (electrodes FT7 and F7) and from the right temporal lobes (electrodes FT8 and F8) were extracted. Then asymmetry values were calculated using the common formula (right-left)/(right + left) (e.g.^58^).

### Site selection

**Figure 2:**
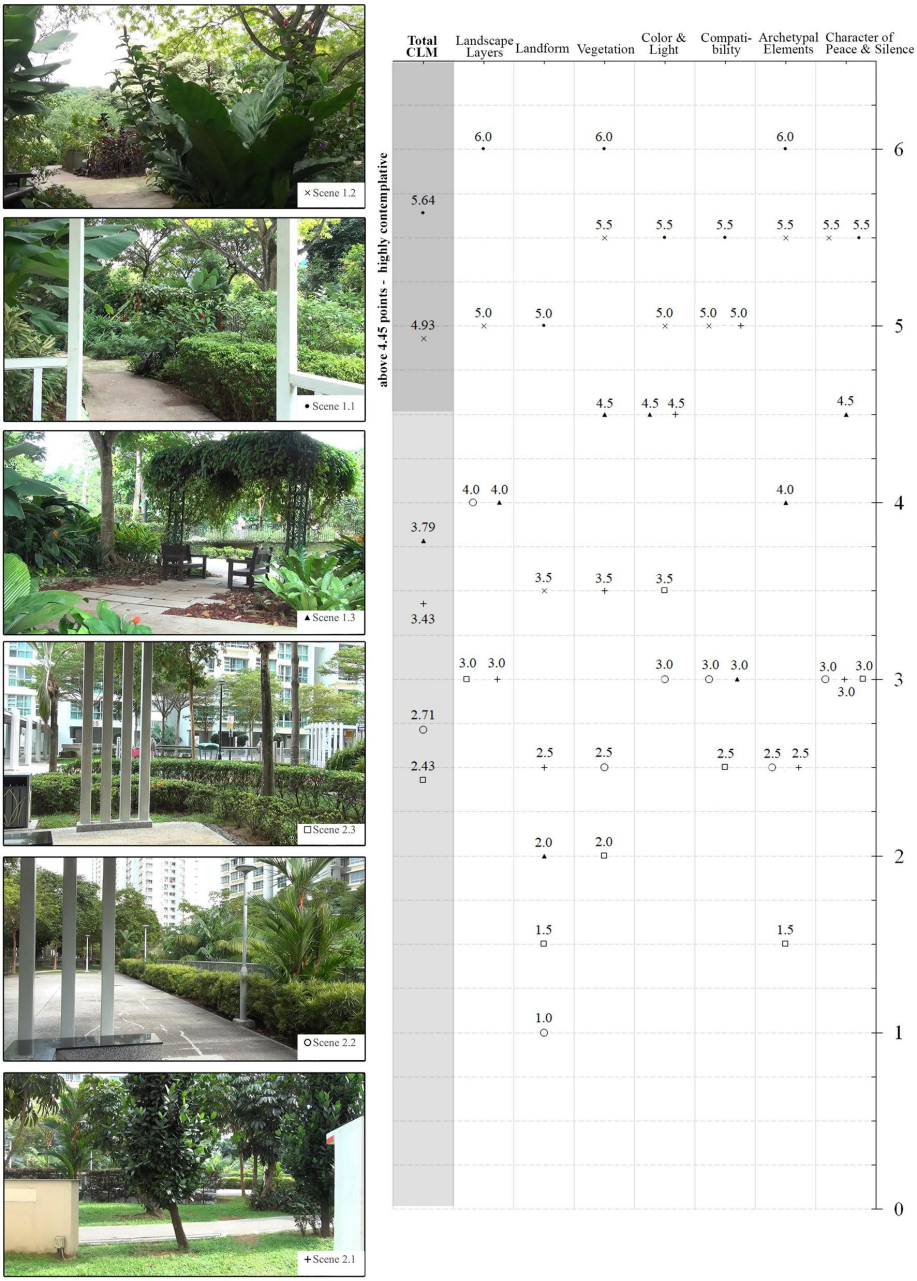
Selected UGS scenes ranking, with CLM scores showing range of different scores (for both total and sub-scales).

Six urban green spaces scenes were selected at two locations in Singapore. Each scene included different natural and built elements and composition types and had previously been annotated by four landscape architecture experts, blinded to hypothesis, using the CLM. Landscape scenes with their scores are presented in the Fig. 2.

Three of the six scenes (S1.1, S1.2 and S1.3) were located within a Therapeutic Garden—a part of a larger urban park named HortPark. This site was selected deliberately due to previous research showing health benefits of contact with nature conducted there, including therapeutic horticulture sessions to promote wellbeing^59^ and reduction of depressive mood among healthy individuals^60^ and depressed patients^61^. Two scenes selected at the Therapeutic Garden (S1.1 and S1.2) scored above the 4.45 point in CLM—the threshold indicating highly contemplative scene, according to previous study^34^. One scene within that garden (S1.3) was selected to represent below-threshold score (3.78 points). S1.1, S1.2 and S1.3 contained unique combinations of physical attributes and had relatively different CLM scores for each of the seven features (see Fig. 2). For example, S1.2 was the only site with a full score in the component *Layers of the Landscape* due to the far distance view over the hills, and view towards the sky wasn’t canopied there. On the contrary, S1.3 was the only one overlooking main constructed walking path with a relatively lower scores in *Landform* and *Compatibility*.

Other scenes (S2.1, S2.2 and S2.3) were located within the roof garden in a public residential estate called Casa Clementi. This site represents a contemporary style of design of public neighbourhoods (developed by the Housing Development Board or HDB)—places where approximately 80% of Singaporeans live^62^. HDB estates are open to public and the green spaces within these estates are an important part of the city green infrastructure^63^. Scenes selected in this location, like the ones in Therapeutic Garden, vary in terms of CLM scoring. On average they have fewer contemplative values, due to the built-up elements and tall buildings dominating almost every view, which limits the reorientation of the viewer from city to nature- like landscape. The highest CLM score here was 3.35 points, at site S2.3 which was overlooking playground and manicured greenery partly covering the façade of the building. The lowest CLM score (2.4 points) was at the view from the void deck of one of the blocks (see Fig. 2).

### Procedures

The experiment consisted of three sessions, one in the laboratory (first one) within the university premises and two remaining ones in the naturalistic settings in the urban green spaces. Data was collected between March 2019 and September 2020, during morning or late afternoon hours of the working week. Experimental sessions were scheduled individually, and the gap between sessions was kept under 30 days.

**Figure 3:**
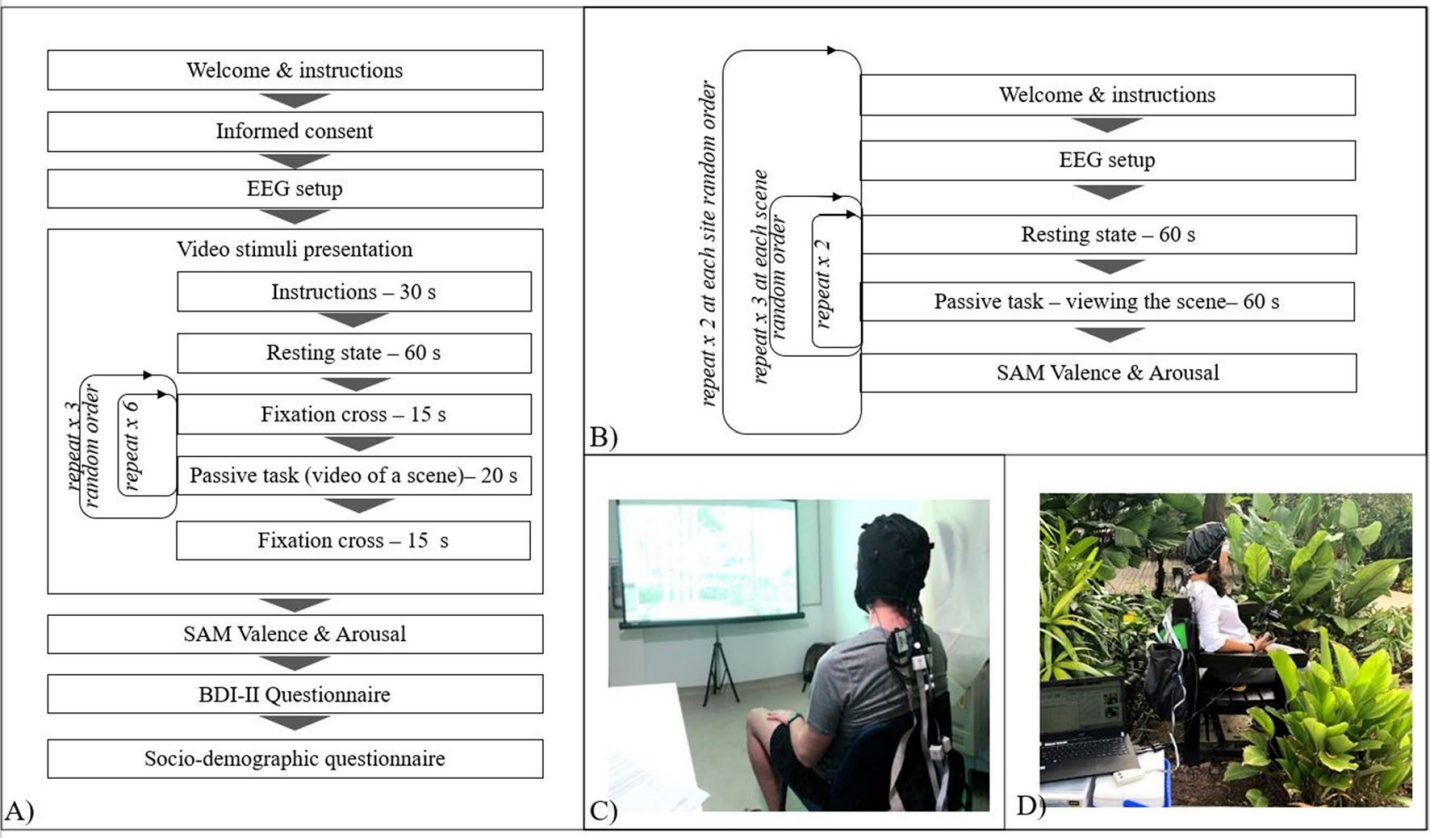
Experimental protocol and setup: (**A**) laboratory setting procedures, (**B**) naturalistic setting procedures, (**C**) experimental setup in the laboratory (participant during viewing of the video), (**D**) experimental setup in the park (participant during resting state).

At the beginning of the first session (in the laboratory), participants signed the informed consent, and had a portable EEG cap adjusted on their head. They were then instructed to sit comfortably on the chair and passively watch the presentation of six fixed-frame videos repeating three times each, in a fully randomized order by the presentation software—Psychopy 3 (2002–2018 Jonathan Peirce, UK^64^). Videos were displayed on a 108 × 178 cm roll-up screen positioned about 200 cm in front of their eyes; projected using the HD29 Darbee Optoma Home Theatre Full HD projector with 1080p (1920 × 1080) screen resolution. There was a 60 s long resting state preceding the video presentations, where participants were looking at a grey, blank screen. Videos were 20 s long (passive task) with a 15 s pause in between, at which time a fixation cross was displayed on the screen (2° of visual angle). The natural sound, as recorded, was also played with the video using standard PC audio speakers placed near the projector, behind the participant’s chair. During the experiment, a daylight-imitating lamp (with a light hue of 5500 K) was turned on (Fig. 3A). After stimuli presentation, the data acquisition cap was removed, and participants were invited to complete the SAM for the individual scenes, BDI-II and Socio-demographic questionnaires. The whole procedure took approximately 50 min. Figure 3C shows the experimental laboratory setup.

During each naturalistic session, one site with three scenes was measured (order randomized^65^). Upon arriving at site, participants were seated on a portable chair facing the selected scene and EEG apparatus was adjusted on their head. Then participants were instructed to put on the white goggles to block the view and relax, while equipment was calibrated, and raw signal recording was initiated. After 1 min recording of the resting state, the participants were asked to remove goggles and passively observe the landscape scene in front for 1 min. Once this was completed, the 1 min resting state with goggle on and 1 min scene watching was repeated for the same scene. This process was repeated for all the three scenes, using the same chair, and the order of the scenes was randomized. After removal of the EEG apparatus, participants were asked to evaluate the scenes with the SAM Valence and Arousal questionnaire (Fig. 3B). The duration of an outdoors session took between 30 and 45 min. Participants were allowed to consume water, but not food, between the scenes. Environmental variables (temperature, humidity, brightness and noise) were recorded with 4-in-1 environment meter (CEM, DT-8820) at each scene at each session to control for confounding variables. The experiment setup in the naturalistic setting is presented in Fig. 3D.

### EEG data processing

Data was processed offline in Brain Analyzer 2 software (Brain Products GmbH, Munich, Germany). The raw signal was filtered with a 50 Hz notch filter, a low-pass at 40 Hz and a high-pass at 0.5 Hz (all were zero phase shift Butterworth filters, order 2). Channels were referenced to an average reference of 16 electrodes and visually inspected for noisy or missing channels. Topographic interpolation of noisy or lost channels was performed where necessary. Ocular artefacts (eye blinks and eye movement) were captured by Independent Component Analysis (ICA) and removed from the data. The signal was epoch time-locked to the video onset (0–20 s). All data underwent Fast Fourier Transform and were output as power. Power values were then averaged over each condition and theta (4–7 Hz), alpha (8–13 Hz) and beta (14–30 Hz) bands power extracted. To compute the wakeful relaxation indices, alpha power values from the viewing condition, from three pairs of frontal electrodes (AFp1-AFp2; AFF5h-AFF6h and F7-F8) was averaged. To compute the mindfulness index, theta power values from the viewing condition, from three pairs of frontal electrodes (AFp1-AFp2; AFF5h-AFF6h and F7-F8) was averaged. To compute the attention restoration index beta power values from the left (electrodes FT7 and F7) and from the right temporal lobe (electrodes FT8 and F8) were extracted. Then asymmetry values were calculated using the common formula (right-left)/(right + left) (e.g.^58^). Separate dataset was created for the laboratory and for the naturalistic setting for each of three power bands.

### Statistical analyses

The CLM scores as well as brain pattern activity scores and self-reported affect scores did not show normal distribution and required non-parametric analyses to account for skewed data. Brain activity data were normalised during data processing to derive alpha, beta and theta scores, and this normalisation did not improve skewness. Spearman's rank-order correlation was run to investigate the relationships between CLM scores and brain pattern activity, as well as self-reported affect scores. A Wilcoxon signed-rank test was conducted to determine differences between outdoor and laboratory datasets between each of the five psychophysiological measurements. We conducted a thorough analysis of environmental conditions, noise, temperature, humidity, brightness, and total mood, and individual situational confounders (alcohol intake in the last 24 h, sleep quality and duration) in relation to the study outcomes. These factors were not linked to the study outcomes and were not included as a part of analysis.

## Results

### Descriptive findings

Due to five participants failing to follow up with all the sessions, only 74 participants (44 females), were included in the study. The average age was 38 ± 17.42 years old. Most of the participants were Chinese. And a large majority had university level of education. The average level of depressive scores, as measured with BDI-II was 8.43 ± 7.31, which indicates overall minimal level of depression. Details about the sample are illustrated in Table 1.

**Table 1:** Recruited sample characteristics.

	Frequency	Percent	Min	Max	Mean	Std. Dev.
**Age**			21	79	38.00	17.42
Youth (18–25)	25	33.8				
Adults (26–44)	26	35.1				
Older adults (45–59)	9	12.2				
Seniors (60+)	14	18.9				
**Severity of depression**						
Total BDI-II			0	36	8.43	7.31
Minimal 0–13 pt	61	82.4				
Mild 14–19 pt	6	8.1				
Moderate 20–28 pt	6	8.1				
Severe 29–63 pt	1	1.4				
**Gender**						
Female	44	59.5				
Male	30	40.5				
**Ethnicity**						
Chinese	49	41.9				
Indian	14	12.0				
Malay	2	1.8				
Others	9	7.7				
**Education**						
Below tertiary	17	23				
Tertiary and above	57	77				
**Vision**						
Corrected	47	40.2				
Normal	27	23.1				
**Hearing**						
Corrected	3	2.6				
Normal	71	60.7				

### Does CLM predict brain pattern activity and self-reported affect? Which specific CLM features contribute to these positive outcomes?

#### CLM scores and Alpha band

There was a significant positive association between Alpha power band recorded outdoors and total CLM score, as well as in the sub-scores Layers of the Landscape, Archetypal Elements, Character of Peace & Silence, and Vegetation, in order of decreasing rho value. Alpha, however, was not significantly related to Color and Light, Landform and Compatibility. There were no significant correlations between Alpha power band and CLM score, or any of the sub-scores in the laboratory settings (Table 2).

**Table 2:** Results of Spearman rank-order correlation.

			CLM	LAY	LAN	VEG	COL	CPB	ARE	CPS
Brain activity (EEG)	Alpha (out)	*r*_*s*_	0.121*	0.145**	0.065	0.121*	0.086	0.062	0.135**	0.130**
		*p*	0.013	0.003	0.179	0.013	0.078	0.200	0.005	0.007
	Alpha (lab)	*r*_*s*_	0.000	0.003	− 0.004	0.000	− 0.001	− 0.010	0.001	0.008
		*p*	0.997	0.954	0.938	0.997	0.980	0.835	0.978	0.875
	Beta (out)	*r*_*s*_	− 0.005	− 0.009	0.003	− 0.005	0.002	− 0.002	− 0.007	− 0.006
		*p*	0.923	0.851	0.952	0.923	0.969	0.973	0.885	0.903
	Beta (lab)	*r*_*s*_	− 0.019	− 0.026	− 0.005	− 0.019	− 0.009	− 0.005	− 0.023	− 0.025
		*p*	0.700	0.586	0.923	0.700	0.856	0.916	0.638	0.606
	Theta (out)	*r*_*s*_	0.143**	0.168**	0.084	0.143**	0.104*	0.086	0.158**	0.149**
		*p*	0.003	0.001	0.083	0.003	0.032	0.075	0.001	0.002
	Theta (lab)	*r*_*s*_	− 0.009	− 0.003	− 0.015	− 0.009	− 0.012	− 0.019	− 0.006	− 0.001
		*p*	0.852	0.949	0.753	0.852	0.802	0.697	0.899	0.980
Self-reported Affect (SAM)	Valence (out)	*r*_*s*_	0.199**	0.237**	0.116*	0.199**	0.151**	0.087	0.221**	0.239**
		*p*	0.000	0.000	0.017	0.000	0.002	0.074	0.000	0.000
	Valence (lab)	*r*_*s*_	0.357**	0.344**	0.288**	0.357**	0.329**	0.221**	0.355**	0.358**
		*p*	0.000	0.000	0.000	0.000	0.000	0.000	0.000	0.000
	Arousal (out)	*r*_*s*_	0.152**	0.156**	0.125*	0.152**	0.143**	0.087	0.156**	0.174**
		*p*	0.002	0.001	0.010	0.002	0.003	0.073	0.001	0.000
	Arousal (lab)	*r*_*s*_	0.148**	0.140**	0.122*	0.148**	0.146**	0.075	0.146**	0.150**
		*p*	0.002	0.004	0.012	0.002	0.003	0.122	0.003	0.002

Significant values are in [bold].

LAY, layers of the landscape; LAN, landform; VEG, vegetation; COL, color and light; CPB, compatibility; ARE, Archetypal elements; CPS, character of peace and silence; *r*_*s*_, Spearman’s rho; CLM, total contemplative landscape model score.

**Correlation is significant at the 0.01 level (2-tailed).

*Correlation is significant at the 0.05 level (2-tailed).

#### CLM scores and Beta band

There were no significant associations between Beta and total CLM score or any of the CLM sub-scores, for both outdoors and laboratory measurements (Table 2).

#### CLM scores and Theta band

Theta scores recorded outdoors were significantly positively associated with total CLM score, as well as the sub-scores Layers of the Landscape, Archetypal Elements, Character of Peace & Silence, Vegetation and Colour & Light, in order of decreasing rho value. Theta, however, was not significantly associated with Landform and Compatibility. There were not significant correlations between Theta power band and CLM score, or any of the sub-scores in the laboratory setting (Table 2).

#### CLM scores and Valence

There were significant positive associations of the SAM Valence recorded outdoors as well as in the laboratory with total CLM, as well as all of the landscape sub-scores, Character of Peace & Silence, Layers of the Landscape, Archetypal Elements, Vegetation, Colour & Light, Landform, and Compatibility. The only exception was for Combability measured outdoors (Table 2).

#### CLM scores and Arousal

There were significant positive associations of SAM Arousal recorded outdoors with total CLM, as well as the sub-scores Character of Peace & Silence, Layers of the Landscape, Archetypal Elements, Vegetation, Colour & Light and Landform, in order of decreasing rho value. SAM Arousal, however, was not significantly associated with Compatibility scores. At the laboratory setting, there were significant positive associations with total CLM, as well as the sub-scores Character of Peace & Silence, Layers of the Landscape, Vegetation, Archetypal Elements, Colour & Light and Landform, in order of decreasing rho value. SAM Arousal, however, was not significantly associated with Compatibility scores (Table 2).

### What are the similarities and differences in outcomes measured in laboratory and outdoors?

A Wilcoxon Signed-Ranks Test indicated that the median Alpha, Beta and Theta values, as well as SAM Arousal scores were significantly lower in the laboratory compared to the naturalistic setting (Table 3). There was no statistically significant difference between the median SAM Valence scores from the laboratory and the outdoors (p = 0.22, Table 3).

**Table 3:** Results of Wilcoxon Signed-Ranks Tests performed to explore differences between laboratory and outdoor setting.

	Mean	Median	Test statistics	
			*Z*	*p*
Alpha out	6.32	4.64	53,504.00	0.032*
Alpha lab	9.01	4.59		
Beta out	0.19	0.20	35,952.00	< 0.005*
Beta lab	0.08	0.11		
Theta out	15.16	11.29	37,517.00	< 0.005*
Theta lab	14.52	8.05		
Valence out	0.92	1.00	13,793.00	0.215
Valence lab	0.86	1.00		
Arousal out	0.08	0.00	12,139.00	< 0.005*
Arousal lab	− 0.26	0.00		

*Denotes statistically significant difference.

## Discussion

The primary goal of this study was to explore the CLM and its specific features association with positive affect and brain activity consistent with relaxation, attention restoration and mindfulness. The secondary goal was to compare these associations in the laboratory and naturalistic outdoor setting.

The findings of this study show that naturalistic exposure to landscapes with higher CLM scores is associated with greater frontal alpha and theta activity, indicative of higher mindfulness^46,47^ and wakeful relaxation^39^. This association was only observed when participants were exposed to naturalistic settings and was not observed while video exposures in the laboratory. These findings are important for the specificity of urban living characterized by overload of stimulation leading to cognitive strain^1,9^—exposed to the highly contemplative landscape, brain activation can simply slow down through increased frontal Alpha and Theta activity, leading to relaxation of that cognitive strain. Higher CLM scores were associated with higher scores for self-reported valence and arousal in both laboratory and outdoor settings indicative of stronger positive emotions, which also are critical for the well-being of the urbanites. Our findings demonstrate that UGS features most strongly associated with brain activity are *Character of Peace and Silence, Layers of the Landscape, Archetypal Elements* and *Vegetation.* Our findings also suggest that there are differences between these associations depending on the exposure setting i.e., the associations are stronger in the outdoor setting as compared to the laboratory setting (Table 2).

CLM score was not associated with Beta temporal asymmetry linked with the Attention Restoration. This may be due to Beta being a high frequency powerband (14–30 Hz), and unlike lower frequency bands (Alpha 8–13 Hz and Theta 4–7 Hz), it is associated with cognitive performance, solving of the tasks and information processing. It seems that this pattern may not be achieved through passive observation of landscapes, as opposed to activities performed in nature. Previous studies with self-reported measures of attention restoration, called Restorative Outcome Scale (ROS^66^), found effect on attention restoration after 30 min–2 h of walking in the park or forest, and not after passive exposure^67,68^. Here we used passive exposure of short duration which might have been insufficient to induce the expected patterns, we also used objective (not self-reported) measures). Interestingly, it was previously found that level of tranquillity (calmness, serenity, peacefulness) of the scene is a major component of the attention restoration theory^69^ but also of relaxation^70^–it then may be possible that what environmental psychologists refer to as Attention Restoration has more to do with the slow brain frequencies rather than Beta waves, associated with attention and cognitive processing. More research is required to unravel the neuroelectric signatures of Attention Restoration as defined by the Attention Restoration Theory.

### Features of urban landscapes and participant outcomes

The landscape features mainly contributing to increased frontal Alpha activity pattern associated with the Wakeful Relaxation, appeared to be *Layers of the Landscape, Archetypal Elements, Character of Peace and Silence* and *Vegetation* respectively (Table 2). The observed significant associations were positive, meaning that higher scores within these landscape categories the greater Alpha power in the frontal cortex. Landscape scenes scoring high in *Layers of the Landscape* category are characterized by a long-distance view and visibility of fore, middle and background within the view. Looking afar has been associated with the psychological comfort and sense of personal freedom; physical distance of the observer may create a sense of psychological distance–seeing things from afar, without overly focusing on details. In other words, seeing a “bigger picture” or sense of “being away”^71^. This can lead to stress reduction through distracting from rumination and fostering contemplation^72^, and aligns with the environmental psychology *Prospect-Refuge* theory which posits that humans derive feelings of safety and pleasure from exposure to environments offering both far-away views and a sense of enclosure^73^. Presence of the *Archetypal Elements* in the landscape also was highly correlated with the Wakeful Relaxation. Such elements in our experimental, settings were single tree, forest and path. It seems that the explicit presence of these objects that, according to Jungian psychoanalysis evoke a subconscious emotional response in all humans, corresponded with relaxation patterns^31,74^. In the traditional Visual Resource Management landscape quality assessment one of the assessment categories was “scarcity”—present of distinct and rare elements, which makes the scene more valuable^28^. Perhaps the presence of *Archetypal Elements* in the scene induced the relaxation pattern in the brain because of the way that they stand out from the landscape, however the specific mechanisms of that being the case are unknown, and further research would be required. Moreover, it is unknown whether other *Archetypal Elements* such as waterfall or stone, not present in our experimental settings would also correlate with the frontal Alpha power. High CLM scores for *Character of Peace and Silence* were also highly related to Wakeful Relaxation, which is not surprising, as it directly corresponds to spaces for resting, comfort and solitude. Higher scores for *Vegetation* (more species diversity, plants looking spontaneous, natural and changing with time) was positively associated with the relaxation patterns in the brain. This observation can be explained by the Biophilia hypothesis, according to which humans feel at home surrounded by unthreatening nature, as well as range of experimental studies reporting less stress and more positive affective response while looking at plants vs looking at other objects^75^. *Color and Light, Compatibility* of the design and *Landform* scores of the scenes were not found to be associated with the frontal Alpha power oscillations. One of the potential reasons could be that the other features of landscapes elicited stronger responses compared to these features, thus reducing the impact that these characteristics had on brain activity. Further research is required where these features are tested in isolation, in the absence of other, perhaps more influential features.

Landscape features mainly contributing to the frontal Theta activity associated with the Mindfulness state, were again: *Layers of the Landscape, Archetypal Elements, Character of Peace & Silence* and *Vegetation*. The explanation for this would be consistent with the previous discussion. Aside from these, a feature that contributed less, but still significantly was *Colour & Light*. Mindfulness, by its definition is associated with being attentive to the present moment, and lack of interpretation or judgement of the experienced phenomena, it involves attention and processing, it is then more than relaxation^48^. *Color & Light* category of CLM is the one involving the motion and feeling that scenery one perceives is alive (passage of the sun across the sky, moving shades of leaves on the ground), thus, being attentive to these subtle changes in the space can stimulate the Mindfulness, but not quite the Wakeful Relaxation, as results of Alpha analysis suggests. *Landform* and *Compatibility* were two features, which did not significantly predict the Mindfulness pattern. It means that the brain may not “need” to detect the undulating topography or diverse skyline or explicit spatial harmony in order to trigger that specific pattern of brain activity.

The contemplative landscapes feature most strongly correlating with the self-reported Valence and Arousal scores was the *Character of Peace and Silence*, suggesting that the higher level of that feature as recognized by experts corresponded with the participants’ self-reported positive affect. Moreover, participants reported high SAM Valence of landscape scenes with explicit presence of *Archetypal Element(s)* and scenes with naturally looking, diverse *Vegetation*. Interestingly, the SAM Valence and Arousal recorded outdoors did not significantly correlate with the *Compatibility* scores. *Compatibility* of a landscape scene, among its other characteristics, depends on the scale, form and composition of the objects within in the view^30^. In the case of the photographs and videos the viewing frame is limited by distinct edges while in the actual park there is no such sharp edge of the view, therefore the *Compatibility* of the view may have been more difficult to grasp by participants—hence the affect associated with the view may be more difficult to assess within this specific subscale.

### Naturalistic and laboratory setting

Our findings suggest the possible weakness of study designs which employ laboratory-only results highlighted in previous research. According to our results, the brainwave oscillations as well as the self-reported Arousal scores were different during the laboratory and outdoor experience. Only the self-reported Valence scores were comparable between the two settings, which corroborates previous research in landscape preference and perceived restorativeness^76,77^. This suggests that the self-reports about the emotional response to landscape settings, collected indoors, based on the photographic or videographic representations can well represent the Valence of emotions (characterized as pleasantness/"good"-ness or averseness/"bad"-ness of a perceived emotion), that would be experienced in the naturalistic setting. In the case of affective Arousal (which corresponds to the intensity of the emotion), it tends to be higher during the exposition to a naturalistic setting than to the photo representation. The above findings are likely related to the immersivity of each experience, looking at the picture or watching a movie is not providing the same level of immersiveness as when being present in the real site. However, people may be able to recognize the landscape from the picture and imagine how they would feel like being there aka, what emotions (positive or negative) they would carry towards the scene, hence they are capable of scoring the scene representation in terms of Valence as they would be when in a real place. This finding can be particularly useful in the conceptual, participatory or competition phases of new UGS developments—if the visualisations of the UGS the proposal have high contemplative values they will likely induce more positive emotional response in raters. But their self-reported Arousal as well as the brainwave scores seem to not be so comparable between the laboratory and naturalistc setting. Future studies could explore more immersive methods of laboratory-based experiments (e.g., 3-dimensional virtual reality) and compare them with in-situ exposure.

The findings of this study are correlational in their nature, therefore should be interpreted with caution. Due to a relatively small number of assessed scenes and lack of representation of other landscape elements described in CLM, assessment of individual features of landscapes in absence of other features was not possible. Due to the recruitment methodology and a relatively small sample size, the sample is not representative of the local population, hence generalisability of findings cannot be ascertained. Also, only the landscapes typical for Singaporean UGS were considered. The generalizability of the findings to other climatic and geographical as well as the urban morphology contexts remains unknown, leaving the room for more research in other countries. Finally, time of stimulation was different in the laboratory (3 × 20 s) and outdoor (2 × 60 s) conditions. There is no gold standard protocol for urban landscape exposure in the naturalistic and outdoor settings. These parameters were selected based on the environmental conditions in Singapore and based on the previous experiences with similar settings and expertise of the study team. To avoid habituation bias and participant fatigue, participants were exposed to 3 shorter stimulations, in the indoor condition. To reduce walking in hot and humid environmental conditions, with the EEG equipment, participants were exposed to 2 longer lasting stimulations outdoors. Due to these methodological differences, comparison of indoor and outdoor stimulations should be interpreted with caution.

## Conclusions

The study findings can help guide planning and design of the UGS with consideration to mental health and well-being of visitors, e.g., therapeutic gardens. CLM can be a useful tool to highlight the existing healthy UGS exposures and design new ones, filling the gap in knowledge about the quality aspects of the UGS design for health. Our study expands on the notion that the quality of elements within the perceived scene is linked to the self-reported affect as well as subconscious brain activity. Our findings suggest that UGS that offer visibility of the *Layers of the Landscape*, contain predominant *Character of Peace and Silence*, with presence of the *Archetypal Elements* and diverse, naturalistic *Vegetation* could be the most valuable from the standpoint of visitors’ wellbeing. Examples of strategies to include in design are:

- opening the views to far-away scenery so that the visitor can see both nearby and faraway objects;
- emphasizing visibility of the aerial perspective, where faraway objects are perceived bluer and more blurred due to volume of the air between them and the eye of the observer;
- creating visual and noise buffers dividing the garden from the city environment, planning for the comfortable seating for solitary resting;
- highlighting the existing archetypal element with design so that it dominates the view (e.g. by clearing the surrounding of the solitary tree to make its silhouette more distinct).
- incorporating more naturalistic planting plans (seemingly planted by nature) including spontaneous and diverse vegetation that displays seasonal and diurnal changes.

Historically, UGS have been envisioned as public spaces for urban populations to seek fresh air and respite, yet the current literature is limited in informing the specific guidelines for design for mental health promotion. Urban planning and design fields should recognise the tremendous mental health benefits that may be harvested from contact with adequately designed and maintained green spaces and include strategies for targeted psychophysiological responses in UGS planning in the national mental health promotion strategies.

## Data Availability

The datasets generated during the current study are available from the corresponding author on reasonable request.
